# Fairness-Aware Resource Allocation in Multi-Hop Wireless Powered Communication Networks with User Cooperation

**DOI:** 10.3390/s18061890

**Published:** 2018-06-08

**Authors:** Ming Lei, Xingjun Zhang, Han Ding, Bocheng Yu

**Affiliations:** Department of Computer Science and Technology, Xi’an Jiaotong University, Xi’an 710049, China; leiming87@stu.xjtu.edu.cn (M.L.); dinghan@xjtu.edu.cn (H.D.); bochengyu@stu.xjtu.edu.cn (B.Y.)

**Keywords:** user cooperation, approximate algorithm, WPCN, max-min throughput problem

## Abstract

In wireless powered communication networks (WPCNs), the harvested energy varies greatly among user nodes (UNs), resulting in throughput unfairness. Since the harvested energy is limited, each UN must strategically allocate the energy used for forwarding the other nodes’ information and for transmitting its own information, which further aggravates the global unfairness in terms of throughput. In this paper, we leverage user cooperation in multi-hop transmission to improve the throughput fairness. We formulate the fairness problem as the max-min throughput with resource allocation, which is NP-hard. We design an approximate algorithm to address this problem. The theoretical proof and the simulation results both show that the proposed algorithm provides tight upper and lower bounds for the optimal solution. Compared with the benchmark methods, our proposed method significantly enhances the throughput fairness for WPCNs.

## 1. Introduction

Wireless sensor networks (WSNs) have developed rapidly due to their ubiquitous applications in a wide range of areas, such as military, transportation, and disaster rescue [1,2,3,4]. In conventional WSNs, sensor nodes are powered by batteries and may not be recharged after deployment. As a result, the network lifetime is severely restricted due to the limited energy provided by the on-board battery. As a promising solution, energy harvesting technologies can combat this power limitation by collecting energy via solar or electromagnetic signals [5,6]. As a representative technology, wireless power transfer (WPT) has several advantages, including a long transmission range and controllable transmission pattern. With the support of WPT, the applications of WPT-based energy harvesting can be divided into two branches. The first branch is the simultaneous wireless information and power transfer [7,8,9], where harvesting energy and information transmission are realized over the separated radio frequency signals simultaneously. The second branch is the wireless powered communication networks (WPCNs). Each user node (UN) in a WPCN harvests energy from an energy transmitter (ET) in the downlink and uses the energy to transmit information in the uplink. Compared to conventional WSNs, WPCNs have a prolonged operating lifetime and provide stable energy to UNs, i.e., sensor nodes [10]. 

However, the throughput fairness in WPCNs remains challenging due to the diversity of the nodes’ physical channel states. In contrast to a stable, on-board, battery energy supply, radio-frequency-based WPT is closely related to the different channel state of each UN. The channel states among UNs are highly diverse due to their distributed locations in WPCNs. The UNs that are further from the ET receive less energy than those closer to the ET. Consequently, the “far” UNs transmit less data than the “near” UNs. Such a “near-far” effect leads to throughput performance unfairness [11].

Prior works have attempted to address this problem in two ways: Equalization-based and cooperation-based approaches. The former type of approach aims to equally balance the throughput by controlling the energy distribution, for example, signal beamforming [12], or by metric-tuning, such as the common throughput [11] and weighted sum-rate [13]. These approaches, however, suffer from either costly hardware, e.g., multi-antenna ET, or strong dependence on the link state between the UNs and ET, as well as between the UNs and the information receiver. On the other hand, the cooperation-based approach establishes cooperation among several UNs to achieve a “win-win” result by designing routine selection, power control, or time allocation mechanisms. In addition to the additional cost and complexity of deployment, dedicated designs that comprehensively combine the above mechanisms are difficult to achieve. Meanwhile, prior works focus on single transmission and fixed paths between source-destination pairs; they do not consider user cooperation in multi-hop transmissions, which is essential for achieving fairness in cooperative WPCNs. In [14], they tested the user cooperation between two UNs and proved that it helped to improve the throughput fairness in a simple two-hop WPCN. However, they assumed that only two UNs transmitted information in the WPCN. In actual multi-user networks, the reasonable transmission path for improving throughput fairness cannot be ignored. 

In this paper, we consider user cooperation in the multi-hop WPCN and find the optimal transmission path in the max-min throughput (MMT) problem. We further formulate this problem as a mixed-integer nonlinear programming (MINLP) model, which is NP-hard and subject to time allocation, energy harvesting, and traffic conservation constraints. The problem cannot be solved directly by existing methods. We design an approximate algorithm, namely, new max-min throughput algorithm (NE-MMT), where the power control, time allocation, and link scheduling are jointly optimized. We present the tight upper and lower bounds of the optimal solution. The extensive simulation results show that NE-MMT can significantly enhance the throughput fairness of WPCNs.

The remainder of the paper is organized as follows. In Section 2, we describe related works. In Section 3, we describe the system model and the MMT problem formulation. In Section 4, we introduce our algorithm for solving the problem. In Section 5, numerical simulations, the performance analysis of our method, and the proposed algorithm are given. Section 6 concludes the paper. 

## 2. Related Work

Methods to improve fairness to combat the “near-far” effect in WPCNs are widely researched. Multi-antenna ET has been proposed to perform the WPT, with the energy beamforming during energy harvesting [12]. In [13], the weighted sum-rate maximization problem was considered to incorporate fairness based on energy beamforming. In [15], energy beamforming was optimally designed for a WPCN in which multiple antennas were equipped at the ET and user cooperation between two UNs was considered. Due to the complexity and high cost of multi-antenna ET deployment, some solutions have considered resource allocation to balance the throughput among UNs in the single-antenna ET case.

In [11], the authors defined a performance metric called common throughput to evaluate the constraint where all UNs were assigned equal throughput by time allocation, regardless of their locations, to address the double “near-far” effect. Based on the same system model in [11], the authors considered the circuit power dissipation, which was not negligible, especially for the WPT. They also proposed a low-complexity, fixed-point, iteration algorithm for the MMT problem in [16]. Because cooperation is an efficient method to improve fairness in wireless powered networks, energy and transmission cooperation have been widely studied [17,18,19,20]. In [21], they considered cooperation in terms of energy, rather than information, and solved an online optimization problem to focus on the long-term weighted throughput performance. In [22], they proposed a novel energy cooperative framework in which the “far” UNs can harvest energy continuously by overhearing signals sent by nearer UNs to the destination node before its information transmission. This cooperative protocol can significantly improve user fairness. In addition to energy cooperation, there has been some research in transmission cooperation. In [20], a “harvest-then-cooperate” protocol was proposed in which the UNs and their dedicated relay nodes harvest energy and then cooperatively work to transmit the information of the UNs to improve the fairness. The relay selection problem, based on the user and relay cooperation, was studied in [23]. The authors maximized the capacity under an energy transfer constraint. In [24], they proposed a Nash bargaining approach to achieve optimal information transmission efficiency of source-destination pairs, with multiple source-destination pairs and one relay. However, setting dedicated relay nodes adds additional costs and increases deployment complexity.

User cooperation is another method to improve fairness performance. User cooperation helps the UNs that harvested more energy to relay the information of the UNs that have less energy or poorer transmission channel states [14]. In [25], they presented a new pricing strategy to motivate one UN, with more energy, to sell its excess energy to help another UN, with less energy, complete a uplink information transfer. The “relay” UN placement and the UN’s communication mode selection problems were also discussed. In [26], the paper considered two communication groups that cooperated via the WPT and time sharing to fulfill their expected information delivery and achieve a “win-win” collaboration. In [27], they presented a new user cooperation method where a pair of wireless powered UNs first exchanged their independent messages with each other and then transmitted jointly to the destination node, enhancing the throughput fairness in WPCNs. In [28], they considered implementing energy-efficient information transfer to mitigate the “near-far” effect. They proposed an optimized transmission protocol to maximize the sum-throughput and chose an appropriate device-to-device communication mode.

In user cooperation, the forwarding UNs must sacrifice harvested energy and transmission time to improve the throughput of the UNs with less energy. If a UN forwards a large amount of information, then the UN needs to spend more time to harvest energy. However, more time spent harvesting energy may lead to less time for transmission for a given time length because UNs cannot simultaneously harvest energy and transmit information in a half duplex [29,30]. Therefore, in the WPCN, the UNs must select a reasonable forwarding UN. Improving fairness performance requires a combination of reasonable routine selection, power control, and time allocation. 

To the best of our knowledge, all the works above improve the fairness via power control and time allocation based on a fixed transmission path and do not consider multi-hop transmission based on user cooperation. In [31], the letter focused on fairness-aware power and time allocation in the WPCN under the “harvest-then-transmit” protocol in which downlink wireless energy transfer was implemented first and, then, uplink wireless information transfer occurred in a spectrum-sharing fashion. They aimed to achieve the rate fairness of all UNs under three fairness criteria: Max-min, proportional, and harmonic fairness. In [32], they studied fairness among UNs and presented a time resource allocation scheme to maximize the individual energy efficiency of each UN based on the max-min criterion. However, they considered the single transmission method without user cooperation. 

## 3. System Model and Problem Formulation

**Figure 1:**
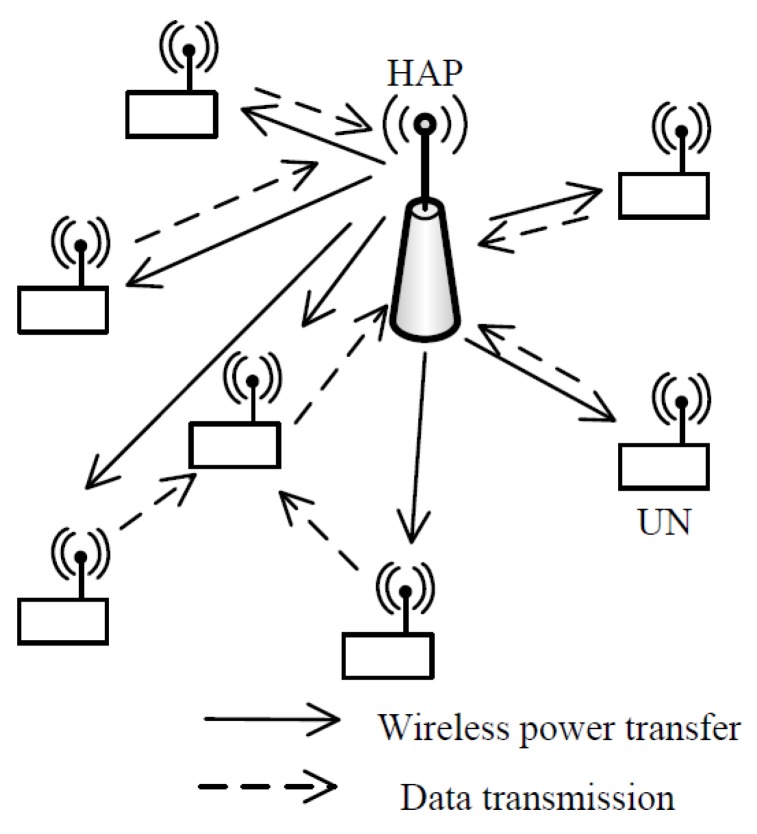
Model of a multi-hop wireless powered communication network.

We consider a WPCN consisting of a hybrid access point (HAP), denoted by H, and a set of UNs, ℕ={1,2,…,N}, without fixed energy sources, as shown in Figure 1. According to the “harvest-then-transmit” protocol, the HAP provides wireless power to all the UNs in the downlink; then, all UNs use the harvested energy to transmit information to the HAP. Due to the “near-far” effect, the UNs close to the HAP obtain more energy than those far from the HAP. More importantly, these “far” UNs consume more energy to transmit information due to the poorer channel quality. Therefore, we consider multi-hop transmission to help the “far” UNs to transmit information.

Before a time block starts, the HAP first uses the algorithm mentioned later to calculate the information, such as forwarding path, transmission time, etc. Then, the HAP broadcasts the information to the UNs. Based on the information, each UN controls its operation in the time block. Since the energy consumption of receiving the controlling information is much less than that of transmission information in the time block, its energy cost is ignored [14,25,26,27,32].

In the time block, the UNs harvest energy from the HAP during the downlink, which is denoted by $t_{0}$. Let $L_{Hi}$ denote the link formed between the HAP and the UN, i, during the downlink. The downlink power gain of link, $L_{Hi}$, is denoted by $h_{Hi}$. We use $P_{0}$ to denote the transmission power of the HAP. The available harvesting energy of the UN, i, is given by the following equation [11]:

(1) $$E_{i}=\zeta P_{0}h_{Hi}t_{0},$$

where ζ represents the power conversion efficiency, which is generally 50–70%. Each UN starts to transmit its information through its output links after it receives the harvested energy. Receiving information and transmitting energy simultaneously causes self-interference at the HAP; thus, the HAP acts only as a sink in the uplink and does not provide wireless power.

We denote the set of all UNs and the HAP as V and use $L_{ij}$ to denote the link formed between the node, i, and the node, j, in the uplink, where ∀i∈V/H and ∀j∈V. The uplink power gain of the link, $L_{ij}$, is denoted by $g_{ij}$. In the transmission uplink, the link may be active for forwarding information only if the quality of the link, $L_{ij}$, is better than that of the link, $L_{iH}$. Otherwise, the link, $L_{ij}$, is inactive and the UN, i, transmits information to the HAP directly due to the limited harvested energy. Links that may be active are called feasible links. The set of all feasible links is denoted by L. We introduce the binary variable, $U_{ij}$, to indicate whether the feasible link, $L_{ij}$, is active in the uplink, $L_{ij}\in L$. If the link, $L_{ij}$, is active, then $U_{ij}$ is one; otherwise, it is zero. When the link, $L_{ij}$, is active, its reverse link must be silent in the uplink. Thus, we have:

(2) $$1\geq U_{ji}+U_{ij},\;\forall L_{ij}\in L$$

Let $t_{ij}$ denote the transmission time of the link, $L_{ij}$. For the convenience of this discussion, we normalize a length of one time block into unit time. We can express the time constraint as:

(3) $$\sum_{L_{ij}\in L}t_{ij}+t_{0}=1,$$

(4) $$t_{ij}\leq U_{ij},\;\forall L_{ij}\in L.$$

where Equation (3) guarantees that the sum of the time for harvesting energy and the time for transmission information does not exceed the length of the time block and then Equation (4) ensures that the transmission time of one feasible link is zero when the link is inactive in the time block. We use $P_{i}$, which takes a value in $\left[0,P_{max}\right]$, to denote the transmission power of the UN, i. During the transmission phase, the energy consumption also includes non-ideal circuit energy consumption, such as sampling and signal processing. We use $P_{c}$ to denote the additional circuit loss energy consumption [33]. The transmission energy consumption of the UN, i, is expressed by:

(5) $$E_{ti}=\sum_{L_{ij}\in L}(P_{c}+P_{i})t_{ij},\;\forall i\in \mathbb{N}.$$

Using the time-division multiple access method for transmission avoids co-channel interference, that is, only one link at a time can be active for data transmission. Therefore, the quality of each active link depends on only the corresponding signal-to-noise ratio (SNR). We use $SNR_{ij}$ to denote the SNR of the link, $L_{ij}$. Once the transmission node, i, of the link, $L_{ij}$, determines the level of transmission power, the link capacity, $C_{ij}$, can be calculated by:

(6) $$C_{ij}=Wlog\left(1+SNR_{ij}\right),$$

where $SNR_{ij}=\frac{P_{i}g_{ij}}{W\eta }$, W is the bandwidth, η is the noise power, and log is base 2 logarithm. We use $r_{ij}$ to indicate the amount of information passing through the link, $L_{ij}$, in the time block. Then, in the time, $t_{ij}$, the amount of information transmitted, $r_{ij}$, must satisfy the following capacity constraint:

(7) $$r_{ij}\leq C_{ij}t_{ij},\;\forall L_{ij}\in L.$$

When the receiving node, j, receives data through the link, $L_{ij}$, it consumes a certain amount of energy for the energy dissipation of the radio, which is caused by running the receiver circuitry and is denoted by $E_{elec}$ nJ/bit. The energy consumed by the UN, i, to receive information is expressed as [34]:

(8) $$E_{ri}=E_{elec}\sum_{L_{ui}\in L}r_{ui},\forall i\in \mathbb{N}.$$

With the inequalities (1), (5), and (8), the energy constraint of the UN, i, can be expressed as:

(9) $$E_{ri}+E_{ti}\leq E_{i}.$$

The data generated and forwarded by each UN must be transmitted through multiple output links. We use $f_{i}$ to represent the amount of data generated by the UN, i. Then, the UN, i, must satisfy the traffic constraint:

(10) $$\sum_{L_{ij}\in L}r_{ij}-\sum_{L_{ui}\in L}r_{ui}\geq f_{i},\forall i\in \mathbb{N}.$$

Before the end of the time block, the data of each UN must be fully imported into the HAP; therefore, the HAP must satisfy the following traffic conservation constraint:

(11) $$\sum_{i\in V/H}f_{i}=f_{H}.$$

where $f_{H}$ denotes the receiving data of the HAP in the time block. Constraints (7) and (10) ensure the reliability of each active link. If (7) and (10) hold, then (11) is satisfied; therefore, constraint (11) can be relaxed. Let f denote the minimum value of the transmitted information of the UNs in the time block; then, the amount of transmitted data for each UN is no less than the values in the time block, that is:

(12) $$f_{i}\geq f,\;\forall i\in \mathbb{N}.$$

The MMT problem is an important issue for optimizing throughput fairness. In this paper, we focus on resource allocation in the max-min problem. As a common indicator of throughput fairness in the MMT problem, we adopt the minimum throughput of the UNs as the performance metric [11], i.e., the amount of data, f. With (12), the objective function of the max-min problem can be transformed into maximizing the amount of data, f. The max-min problem is modeled as the MMT, which is:

$$\begin{array}{cc} \mathrm{MMT:}:\max\;f & \\ & s.t.\;\left(1\right)-\left(10\right),\left(12\right)\\ & U_{ij}\in \left\{0,1\right\},r_{ij}\geq 0,f\geq 0,P_{i}\geq 0,t_{0}\geq 0,t_{ij}\geq 0. \end{array}$$

The MMT is a MINLP model. Such programming problems are, generally, NP-hard [35]. The existing methods cannot be used to solve the problem directly. The programming model shows that the main challenges in solving the model are as follows: (1) The multiplication of the nonlinear log function and linear variable appears on the right side of constraint (7) in the MMT; and (2) bi-linear products, such as $P_{i}t_{ij}$, exist. In the next section, we propose converting the model to a mixed-integer linear programming (MILP) model via the piece-wise linear (PWL) method. Based on the PWL method, we propose an approximate algorithm called NE-MMT.

## 4. NE-MMT Approximate Algorithm for the Max-Min Problem

In the MMT, constraint (7) contains the log function, which leads to the main difficulty in solving the problem. We use the PWL method to transform constraint (7) into a linear constraint.

### 4.1. PWL Method to Transform the Nonlinear Function into a Piece-Wise Linear Function

**Figure 2:**
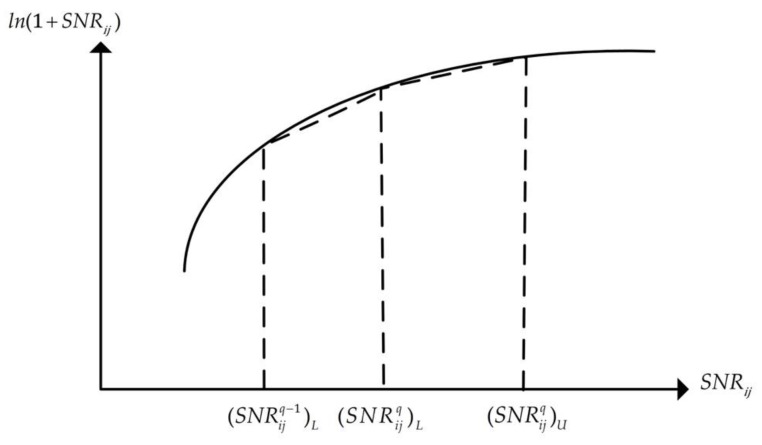
An illustration of linearizing the ln function in the q th interval of $SNR_{ij}$.

We use the proposed PWL method to linearize the log function term [36], as shown in Figure 2. The idea behind the PWL method is to approximate the log curve (base e) by a set of line segments and guarantee that the gap between the piece-wise function and the log function (base e), denoted as the ln function, is less than a threshold. We denote the threshold as γ. For the sake of discussion, we use the following constraint in place of constraint (6):

(13) $$C_{ij}=\frac{W}{ln2}ln\left(1+SNR_{ij}\right)$$

Since the ln function of the corresponding active links, $L_{ij}$, is a function of the $SNR_{ij}$ variable, we segment the interval of $SNR_{ij}$, which is $\left[0,P_{max}g_{ij}/W\eta \right]$, into multiple segments. We use $D_{ij}$ to denote the number of segments in the interval, $\left[0,P_{max}g_{ij}/W\eta \right]$. The qth interval segment is expressed as $\left({\left(SNR_{ij}^{q}\right)}_{L},{\left(SNR_{ij}^{q}\right)}_{U}\right),q\in D_{ij}$. The slope of the qth piece-wise segment corresponding to $SNR_{ij}$ is denoted by $v_{ij}^{q}$ and is expressed as:

(14) $$v_{ij}^{q}=\frac{ln\left(1+{\left(SNR_{ij}^{q}\right)}_{U}\right)-ln\left(1+{\left(SNR_{ij}^{q}\right)}_{L}\right)}{{\left(SNR_{ij}^{q}\right)}_{U}-{\left(SNR_{ij}^{q}\right)}_{L}}$$

Next, we determine the value of $D_{ij}$ via the PWL method, such that the gap between the piece-wise function and the log function is less than the threshold, γ. The process of the PWL method is shown in Algorithm 1.


**Algorithm 1: Piece-wise Linear Method**

Initialization: q=0, $D_{ij}=0$, ${\left(SNR_{ij}^{q}\right)}_{L}=0$.Use Newton’s method to solve the following equation:
$-ln\left(v_{ij}^{q}\right)+v_{ij}^{q}\left(1+{\left(SNR_{ij}^{q}\right)}_{L}\right)-1-ln\left(1+{\left(SNR_{ij}^{q}\right)}_{L}\right)=\gamma .$
Determine the slope $v_{ij}^{q}$.
3.Use Newton’s method to solve Equation (14) to obtain ${\left(SNR_{ij}^{q}\right)}_{U}$If ${\left(SNR_{ij}^{q}\right)}_{U}\geq P_{max}g_{ij}/W\eta ,$ then stop and set ${\left(SNR_{ij}^{q}\right)}_{U}$ and $D_{ij}$ to $P_{max}g_{ij}/W\eta$ and q. Otherwise, continue to step 4.4.Use the equation ${\left(SNR_{ij}^{q}\right)}_{U}={\left(SNR_{ij}^{q+1}\right)}_{L}$ to obtain the value of ${\left(SNR_{ij}^{q+1}\right)}_{L}$. Set q = q+1, return to step 2.


The log function of the variable, $SNR_{ij}$, can be transformed to a linear piece-wise via Algorithm 1. The value of the linear piece-wise function is the lower bound of that of the log function. We convert constraint (7) to the following group of constraints with the log function:

(15) $$r_{ij}\leq \frac{Wt_{ij}}{ln2}\left[v_{ij}^{q}\left(\frac{P_{i}g_{ij}}{W\eta }-{\left(SNR_{ij}^{q}\right)}_{L}\right)+ln\left(1+{\left(SNR_{ij}^{q}\right)}_{L}\right)\right],\forall L_{ij}\in L,q\in D_{ij}$$

After replacing constraint (7) in the MMT with constraint (15), all the terms are linear in the MMT, except for the term $P_{i}t_{ij}$. We use a new variable, $\alpha_{ij}$, to represent the bi-linear product term. The MMT can be transformed into MMT1 as follows:

$$\begin{array}{l} \mathrm{MMT1}:\max\;f\\ \;s.t.\;f_{i}\geq f,\;\forall i\in \mathbb{N}\\ \;1\geq U_{ji}+U_{ij},\;\forall L_{ij}\in L\\ \;\sum_{L_{ij}\in L}t_{ij}+t_{0}=1\\ \;t_{ij}\leq U_{ij},\;\forall L_{ij}\in L\\ \;\sum_{L_{ij}\in L}(P_{c}t_{ij}+\alpha_{ij})+E_{elec}\sum_{L_{ui}\in L}r_{ui}\leq \zeta P_{0}h_{Hi}t_{0}\\ \;\sum_{L_{ij}\in L}r_{ij}-\sum_{L_{ui}\in L}r_{ui}\geq f_{i},i\in V/H\\ \;r_{ij}\leq \frac{W}{ln2}\left[v_{ij}^{q}\left(\frac{\alpha_{ij}g_{ij}}{W\eta }-{\left(SNR_{ij}^{q}\right)}_{L}t_{ij}\right)+t_{ij}ln\left(1+{\left(SNR_{ij}^{q}\right)}_{L}\right)\right],\forall L_{ij}\in L,q\in D_{ij}\\ \;U_{ij}\in \left\{0,1\right\},r_{ij}\geq 0,f\geq 0,\alpha_{ij}\geq 0,t_{0}\geq 0,t_{ij}\geq 0 \end{array}$$

The new model eliminates the variable, $P_{i}$, and is a MILP problem that can be easily solved by existing optimization software, such as Gurobi and CPLEX. We now give the relationship between the solution of the MMT and that of the MMT1.

**Lemma** **1.** *The optimal solution of the MMT1 is a feasible solution of the MMT.*

**Proof.**  The only difference between the MMT and the MMT1 is the capacity constraint. The capacity constraint in the MMT1 is obtained by linearizing the right term of the corresponding constraint in the MMT. For the given values, $P_{i}$ and $t_{ij}$, the value of the right term of the capacity constraint (15) in the MMT1 is no more than that of the capacity constraint (7) in the MMT. The solution space of the variable, $r_{ij}$, in the MMT1 is covered by that in the MMT. Because of the same solution spaces of the other variables, the optimal solution in the MMT must be a feasible solution to the MMT. □

We see from Lemma 1 that the optimal solution in the MMT1 provides a lower bound to that of the MMT. We rewrite the group of capacity constraints in the MMT1 as:

(16) $$r_{ij}\leq \frac{Wt_{ij}}{ln2}\left[v_{ij}^{q}\left(\frac{P_{i}g_{ij}}{W\eta }-{\left(SNR_{ij}^{q}\right)}_{L}\right)+ln\left(1+{\left(SNR_{ij}^{q}\right)}_{L}\right)\right]+\frac{\gamma W}{ln2},\forall L_{ij}\in L,q\in D_{ij}.$$

With the new capacity constraint (16), we build a new model as the MMT2:

$$\begin{array}{l} \mathrm{MMT:}:\max\;f\\ \;s.t.f_{i}\geq f,\;\forall i\in \mathbb{N}\\ \;1\geq U_{ji}+U_{ij},\;\forall L_{ij}\in L\\ \;\sum_{L_{ij}\in L}t_{ij}+t_{0}=1\\ \;t_{ij}\leq U_{ij},\;\forall L_{ij}\in L\\ \;\sum_{L_{ij}\in L}(P_{c}t_{ij}+\alpha_{ij})+E_{elec}\sum_{L_{ui}\in L}r_{ui}\leq \zeta P_{0}h_{Hi}t_{0}\\ \;\sum_{L_{ij}\in L}r_{ij}-\sum_{L_{ui}\in L}r_{ui}\geq f_{i},i\in V/H\\ \;r_{ij}\leq \frac{Wt_{ij}}{ln2}\left[v_{ij}^{q}\left(\frac{P_{i}g_{ij}}{W\eta }-{\left(SNR_{ij}^{q}\right)}_{L}\right)+ln\left(1+{\left(SNR_{ij}^{q}\right)}_{L}\right)\right]+\frac{\gamma W}{ln2},\forall L_{ij}\in L,q\in D_{ij}.\\ \;U_{ij}\in \left\{0,1\right\},r_{ij}\geq 0,f\geq 0,\alpha_{ij}\geq 0,t_{0}\geq 0,\;t_{ij}\geq 0 \end{array}$$

Through a similar proof as that of Lemma 1, we can find the upper bound of the optimal solution of the MMT.

**Lemma** **2.** *The optimal solution of the MMT2 is the upper bound of that of the MMT.*

**Proof.**  The only difference between the models of the MMT and the MMT2 is the use of capacity constraint (7) in the MMT and constraint (16) in the MMT2. The capacity of the link, $L_{ij}$, in the MMT is no more than the upper bound value. Thus, we have:

$$ln\left(1+SNR_{ij}\right)\leq v_{ij}^{q}\left(\frac{P_{i}g_{ij}}{W\eta }-{\left(SNR_{ij}^{q}\right)}_{L}\right)+ln\left(1+{\left(SNR_{ij}^{q}\right)}_{L}\right)+\gamma$$

Since the transmission time of the link, $L_{ij}$, is subject to $1>t_{ij}\geq 0$, we have:

$$\begin{array}{cc} r_{ij}\leq c_{ij}t_{ij}= & \frac{Wt_{ij}}{ln2}\left[v_{ij}^{q}\left(\frac{P_{i}g_{ij}}{W\eta }-{\left(SNR_{ij}^{q}\right)}_{L}\right)+ln\left(1+{\left(SNR_{ij}^{q}\right)}_{L}\right)+\gamma \right]\\ & \;\;\leq \frac{Wt_{ij}}{ln2}\left[v_{ij}^{q}\left(\frac{P_{i}g_{ij}}{W\eta }-{\left(SNR_{ij}^{q}\right)}_{L}\right)+ln\left(1+{\left(SNR_{ij}^{q}\right)}_{L}\right)\right]+\frac{\gamma W}{ln2} \end{array}$$

The solution space of the variable, $r_{ij}$, in the MMT is covered by that in the MMT2, $\forall L_{ij}\in L$. Because of the same solution space for the other variables, the optimal solution of the MMT2 is the upper bound of the optimal solution of the MMT. □

Although we cannot obtain the optimal solution of the problem, we use Lemmas 1 and 2 to determine the boundaries of the optimal solution. However, we do not know how large the gap between the upper and lower bounds is. We use the approximate algorithm in the next section to ensure that the gap is within a specified range.

### 4.2. Approximate Algorithm for the Max-Min Problem

In the MMT, the group of variables, $U_{ij}$, is the unique integer variable set, $\forall L_{ij}\in L$. As the active state of the link, $L_{ij}$, and the transmission power of the transmission node are given, the MMT is transformed into a linear programming model of the traffic and time allocation. We use $\left({\bar{P}}_{i},{\bar{U}}_{ij}\right)$ to denote the case of a determined state of the link, $L_{ij}$, and the chosen transmission power of the UN, i, $\forall i\in V/H,\;\forall L_{ij}\in L.$ Then, for the given case, $\left({\bar{P}}_{i},{\bar{U}}_{ij}\right)$, the MMT is expressed as the MMT$\left({\bar{P}}_{i},{\bar{U}}_{ij}\right)$ as:

MMT$\left({\bar{P}}_{i},{\bar{U}}_{ij}\right):\;\max\;f$

(17) $$s.t.\;f_{i}\geq f,\;\forall i\in \mathbb{N}$$

(18) $$\sum_{L_{ij}\in \bar{L}}t_{ij}+t_{0}=1$$

(19) $$\sum_{L_{ij}\in \bar{L}}(P_{c}+{\bar{P}}_{i})t_{ij}+E_{elec}\sum_{L_{ui}\in \bar{L}}r_{ui}\leq E_{i},\forall i\in \mathbb{N}\;$$

(20) $$r_{ij}\leq {\bar{c}}_{ij}t_{ij},\;\forall L_{ij}\in \bar{L}$$

(21) $$\sum_{L_{ij}\in \bar{L}}r_{ij}-\sum_{L_{ui}\in \bar{L}}r_{ui}\geq f_{i},\forall i\in \mathbb{N}$$

$$r_{ij}\geq 0,f_{i}\geq 0,f\geq 0,t_{ij}\geq 0,t_{0}\geq 0$$

where $\bar{L}$ denotes the set of active links in the given $\left({\bar{P}}_{i},{\bar{U}}_{ij}\right)$ case, and ${\bar{c}}_{ij}$ represents the capacity of the link, $L_{ij}$, in the given case, which can be calculated by (6). We use (15) to obtain the capacity of the link, $L_{ij}$, in the same case and denote it as ${\hat{c}}_{ij}$. If we use the constraints (22) and (23) in place of the capacity constraint (21), the model MMT$\left({\bar{P}}_{i},{\bar{U}}_{ij}\right)$ is transferred into two new models, denoted by MMT-UP$\left({\bar{P}}_{i},{\bar{U}}_{ij}\right)$ with constraint (22) and MMT-DOWN$\left({\bar{P}}_{i},{\bar{U}}_{ij}\right)$ with constraint (23):

(22) $$r_{ij}\leq {\hat{c}}_{ij}t_{ij}+\frac{\gamma W}{ln2},\;\forall L_{ij}\in \bar{L}$$

(23) $$r_{ij}\leq {\hat{c}}_{ij}t_{ij},\;\forall L_{ij}\in \bar{L}$$

According to Lemmas 1 and 2, we obtain the upper and lower bounds of the optimal solution of the MMT$\left({\bar{P}}_{i},{\bar{U}}_{ij}\right)$ by solving the MMT-UP$\left({\bar{P}}_{i},{\bar{U}}_{ij}\right)$ and the MMT-DOWN$\left({\bar{P}}_{i},{\bar{U}}_{ij}\right)$, respectively. We denote the upper and lower bounds in the given $\left({\bar{P}}_{i},{\bar{U}}_{ij}\right)$ case as $\bar{f}$ and $\underline{f}$. The gap between the upper and lower bounds is denoted by Δf, that is, $\Delta f=\bar{f}-\underline{f}$. We find the characteristics of the gap in the given $\left({\bar{P}}_{i},{\bar{U}}_{ij}\right)$ case.

**Lemma** **3.** *In the given*$\left({\bar{P}}_{i},{\bar{U}}_{ij}\right)$*case, the gap*$\Delta f\leq \frac{\gamma W}{ln2}\left|\bar{L}\right|$.

**Proof.**  The proof of Lemma 3 is given in Appendix A. □

Through Lemma 3, we determine the relationship between the upper and lower bounds of the MMT. We use ${\bar{f}}^{*}$ and ${\underline{f}}^{*}$ to represent the upper and lower bounds, which are obtained by solving the MMT2 and the MMT1. The gap between them is denoted by $\Delta f^{*}$.

**Lemma** **4.** *Assume that the number of feasible links is*|L|*; then, the gap between the upper and lower bounds of the optimal solution of the MMT is no less than*$\frac{\gamma W}{ln2}\left|L\right|$*, that is,*$\Delta f^{*}\leq \frac{\gamma W}{ln2}\left|L\right|$.

**Proof.**  We use $\left(t_{ij}^{*},U_{ij}^{*},P_{i}^{*},f^{*},f_{i}^{*},r_{ij}^{*}\right)$ to represent the optimal solution of the MMT. In the given case of $\left(U_{ij}^{*},P_{i}^{*}\right)$, the optimal solutions of the MMT2 and MMT1 are expressed as $\left(\bar{t_{ij}^{*}},U_{ij}^{*},P_{i}^{*},\bar{f^{*}},\bar{f_{i}^{*}},\bar{r_{ij}^{*}}\right)$ and $\left(\underline{t_{ij}^{*}},U_{ij}^{*},P_{i}^{*},\underline{f^{*}},\underline{f_{i}^{*}},\underline{r_{ij}^{*}}\right)$, respectively. According to Lemma 3, $\Delta f^{*}\leq \frac{\gamma W}{ln2}\left|L^{*}\right|$, where $L^{*}$ represents the optimal set of active links. Since $L^{*}\in L$, we obtain $\Delta f^{*}\leq \frac{\gamma W}{ln2}\left|L\right|$. □

According to Lemma 4, the gap between the optimal solution of the original problem and the upper bound or lower bound is no greater than $\Delta f^{*}$. We propose our following NE-MMT approximate algorithm based on Lemma 4.


**Algorithm 2: NE-MMT Algorithm**

Initialization: $\Delta f^{*}$.Solve the following equation: $\;\Delta f^{*}=\frac{\gamma W}{ln2}\left|L\right|$. Determine γ.Use the PWL method to transform the MMT into a MILP model.Solve the MMT-UP or MMT-DOWN with a solver, such as CPLEX. Obtain an approximate solution.


Algorithm 2 produces the upper and lower bounds of the original problem. A smaller value of $\Delta f^{*}$ indicates that the performance of the solution is closer to the optimal solution.

## 5. Experiment and Evaluation

### 5.1. Simulation Setup

In the WPCN, the bandwidth is set to 1 MHz and the noise power, η=−90 dBm. We assume the channel short-term fading is Rayleigh distributed. The channel power gains, $h_{Hj}=10^{-3}\rho_{Hj}^{2}d_{Hj}^{-\alpha },g_{ji}=10^{-3}\rho_{ji}^{2}d_{ji}^{-\alpha }$, ∀i∈V,j∈V/H [6], $\rho_{ij}^{2}$ follow the Rayleigh distribution, $d_{ij}$ denotes the distance between the UN, i, and the UN, j, and α is the path-loss exponent. We assume a 30 dB average signal attenuation at the 1 m reference distance and we set $\Delta f^{*}=0.1$ Kb. All the algorithms are implemented by C++ and GPLEX on a Sony EA37EC notebook with an Intel Core i3 CPU (2.4 GHz) and 4 GB RAM. We run 100 instances of each setup.

We simulate the network throughput fairness performance using our proposed multi-hop transmission method based on user cooperation. We use “MMT2” and “MMT1” to express the upper and lower bounds obtained by our proposed method. Few studies have considered path mechanisms in WPCNs. Thus, we chose two conventional wireless network path mechanisms with user cooperation as performance benchmarks: Random progress (RA), in which the data of the UNs are routed with equal probability through a feasible link; and the greedy method (GR), in which the UNs transmit information by a feasible link based on which receiver node is closest to them. In addition, we also assess the direct transmission method in which each UN transmits its information directly to the HAP without user cooperation [6]. The “without user cooperation” method is the third benchmark used to demonstrate the effect of user cooperation on throughput fairness. In Section 3, the minimum throughput of the UNs is considered as a measure of user fairness. Therefore, we focus on the average minimum throughput of the UNs, that is, the data value, f, as the performance measure in the simulation.

### 5.2. Routing Results for the WPCN

**Figure 3:**
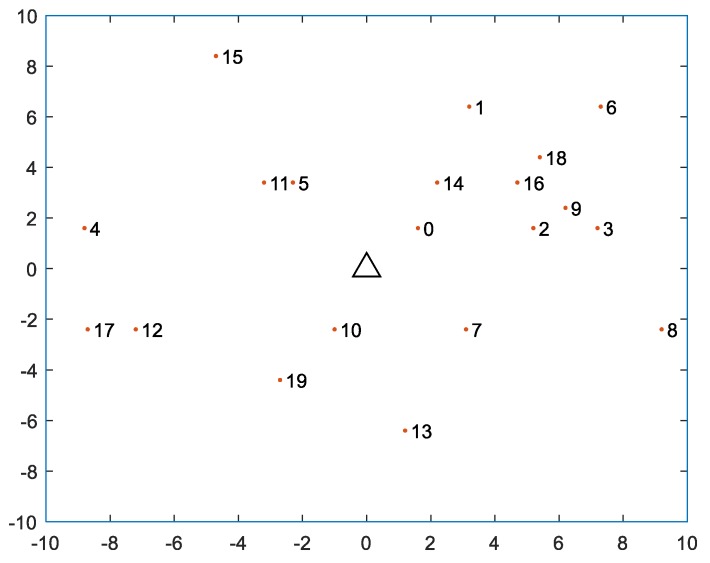
The topology for a 20-node wireless powered communication network.

In this WPCN, we assume that there are |ℕ| = 20 UNs. The topology of the 20-node WPCN is shown in Figure 3. The HAP is at the center point and is denoted by a triangle symbol. The UNs are randomly distributed in the 10 × 10 square area and denoted by the red dots. We assume the transmission power of HAP is 30 dBm. The path-loss exponent is set to 2.

**Figure 4:**
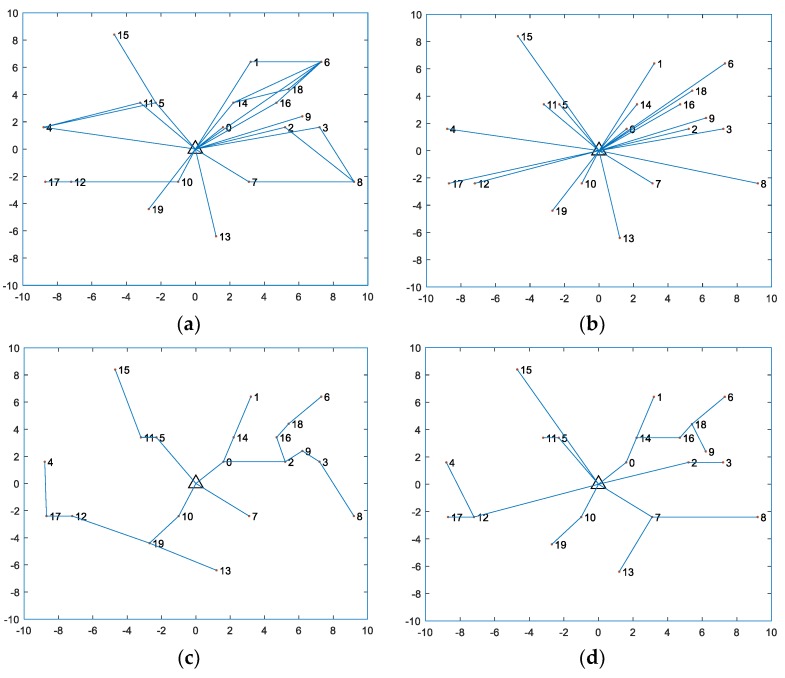
Transmission routing results for the 20-node WPCN. (**a**) Transmission routing result obtained by the proposed method; (**b**) Transmission routing result obtained by the “without user cooperation” method; (**c**) Transmission routing result obtained by the “GR” method; (**d**) Transmission routing result obtained by the “RA” method.

Figure 4 shows the transmission routing results obtained by our proposed method, the RA method, the GR method, and the “without user cooperation” method. As can be seen from Figure 4a, the proposed method can achieve a good balance between transmitting information and energy consumption. As shown in Figure 4a, UN 2 cannot help to forward information generated by UN 9 due to its limited energy. Although UN 4 is closest to UN 17, it transmits its information to UN 11 and UN 5. This is because UN 17, far from the HAP, harvests little energy and has no remaining energy for forwarding other UNs’ information. UN 2, UN 3, and UN 7 are not close to the HAP. They have limited harvested energy from broadcasting signals of the HAP. Therefore, they can only jointly help UN 8 to forward information. 

The routing result obtained by the “without user cooperation” method is shown in Figure 4b. Since the method does not consider user cooperation, the information from UN 15 is transmitted to the HAP over a long distance, which causes the large signal attenuation. The throughput of UN 15 is greatly affected. Since the way to improve the throughput fairness performance is mainly through improving the throughput performance of the UNs far from the HAP, the low throughput of UN 15 becomes a bottleneck in throughput fairness performance. However, the information from UN 15 is forwarded by UN 5 in Figure 4a. Using the proposed method can break the bottleneck.

Figure 4c,d shows the routing results obtained by the “RA” method and the “GR” method. It can be found that UN 17 forwards information from UN 4 in Figure 4c. Although the UN17 is closest to the UN 4, it is very far from the HAP. The UN 17 has a low harvested energy and it is almost impossible to forward information. Thus, the throughput of UN 4 is affected. The information of UN 4 is forwarded by UN 11 by the proposed method in Figure 4a. UN 11, close to the HAP, has a certain amount of residual energy to help UN 4 transmit information. Thus, using the proposed method can improve the throughput fairness. In Figure 4d, the transmission information from UN 6 and UN 9 is transmitted to UN 18. With limited energy, UN 18 can only forward very little information. Therefore, the throughput of UN 6 and UN 9 is very low. Using the proposed method can help UN 6 transmit information to the “relay” nodes, UN 1, UN 14, UN 16, and UN 18. This can improve the throughput of UN 6. 

The minimum throughput corresponding to the routing results in Figure 4a–d is 4.75 Kb, 4.46 Kb, 2.48 Kb, and 0.52 Kb, respectively. Compared with the benchmark methods, the proposed method can improve throughput fairness efficiently. In the next section, we simulate the impact of changes in the parameters on the throughput fairness.

### 5.3. Throughput Fairness for the WPCN

**Figure 5:**
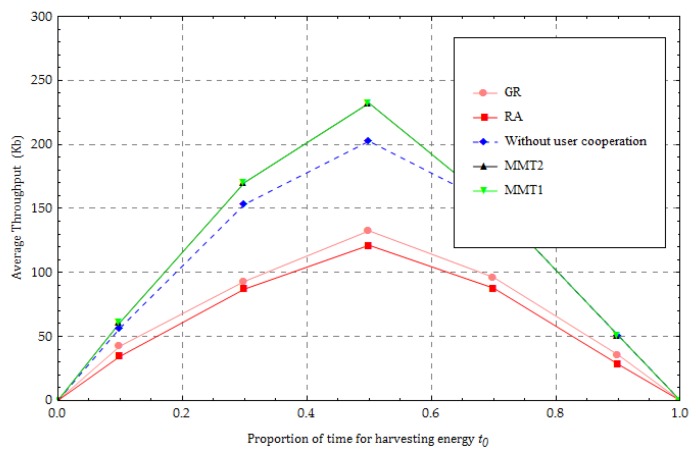
Change in the proportion of time for harvesting energy.

Figure 5 shows the average throughput for various given values of the proportion of time for harvesting energy, with $P_{0}=40$ dBm, α=2, and N=20. It is observed that the average throughput first increases as $t_{0}$ increases from 0 to 0.5. This is because the UNs harvest more energy with increasing $t_{0}$. More harvested energy helps the UNs transmit more information in the uplink. However, as $t_{0}\;$ becomes larger than 0.5, the average throughput decreases due to the reduction of transmission time in the uplink. In addition, using the proposed method can significantly increase the average throughput as $t_{0}$ in the interval [0,0.5]. This is because the UNs close to the HAP obtain more energy and can help the “far” UNs forward information. The throughput of “far” UNs increases with user cooperation. However, the throughput performance when using our proposed method and the “without user cooperation” method is the same as when $t_{0}$ is in the interval [0.7,1]. This is because the UNs harvest enough energy over a long time. The energy can cause the UNs to directly transmit information to the HAP in the uplink. In Figure 5, the gap between the upper bound and lower bound is less than the preset value 0.1 Kb. 

**Figure 6:**
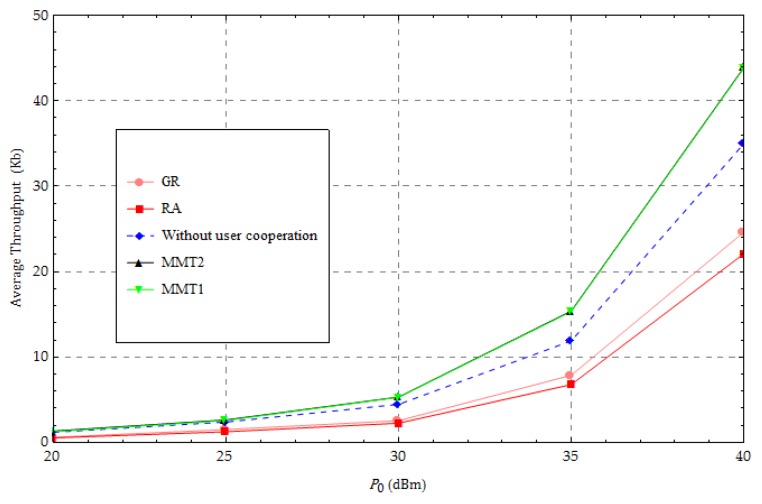
Change in the transmission power of the hybrid access point.

Figure 6 displays the average throughput for various values of transmission power of the HAP, with α=3 and  N=20. The optimized throughput performance tends to increase with $P_{0}$, which is expected because a larger $P_{0}$ provides more energy when harvesting energy, which provides more energy to transmit information. Figure 6 shows that the throughput when using our proposed algorithm or without cooperation is greater than that when using GR or RA. In Figure 6, the average throughput performance when using our proposed method and the “without user cooperation” method is similar to when $P_{0}$ is small. Our proposed method improves the average throughput performance as the intensity of harvested energy increases because more energy can be harvested by UNs and used to forward information as $P_{0}$ increases. When $P_{0}=40$ dBm, our proposed method improves the throughput by 25%, 76%, and 100%, respectively, compared with the other three methods. In Figure 6, the upper bound and lower bound of the optimal solution, which are solved by our algorithm, are close. We can see the gap between the upper bound and lower bound is no more than 0.1 Kb.

**Figure 7:**
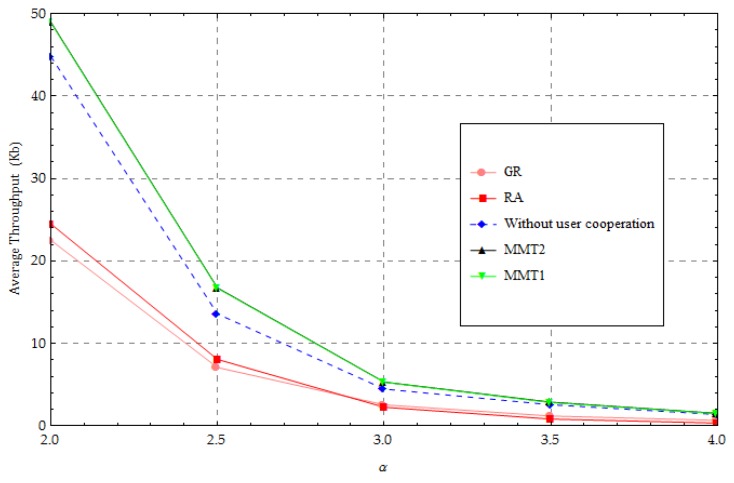
Change in the path-loss exponent.

In the third simulation study, the average throughput performance under the individual path-loss exponent is evaluated using different methods, with $P_{0}=30$ dBm, as shown in Figure 7. As the path-loss exponent decreases, the average throughput is observed to increase rapidly. This is because decreasing the path-loss exponent helps to improve the channels for harvested energy and transmission. When the path-loss exponent is large, the performance of our proposed method is similar to that of the “without user cooperation” method because when the qualities of links are poor, the UNs cannot provide sufficient energy for forwarding information. However, the proposed method is far better than that of the GR and RA methods when the path-loss exponent is large. This is because the traditional methods force some nodes to become “relay” nodes, causing bottlenecks in the average throughput performance of these “relay” nodes. When the path-loss exponent is small, the performance gap of the throughput between the proposed method and the “without user cooperation” method increases. This is because the UNs close to the HAP obtain more energy and have better channel quality as the path-loss exponent decreases. These UNs begin to utilize more energy for forwarding information and help the UNs far from the HAP improve the throughput performance. In addition, with the proposed algorithm we can obtain a small gap in the average throughput performance between the upper and lower bounds of the optimal solution.

**Figure 8:**
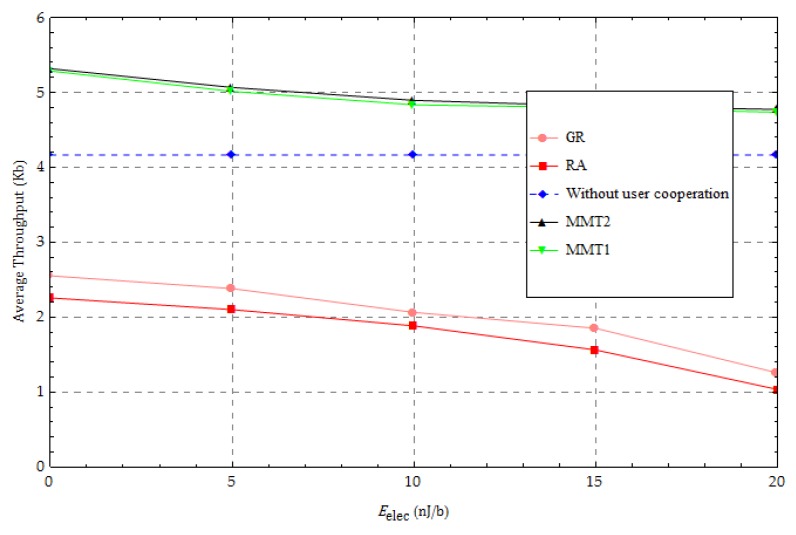
Change in the amount of receiver circuitry.

We show the effect of the energy dissipation of the radio caused by running the receiver circuitry, with $P_{0}=30$ dBm and α=3 in Figure 8. Because the “without user cooperation” method does not include information forwarding, its average throughput performance is not affected by this change in parameter values. We observe that the average throughput performances of both traditional methods and our proposed method decrease as the energy dissipation, $E_{elec}$, increases because a UN needs to allocate more energy to cope with the energy cost of receiving forwarding information as the energy dissipation is increased. When the energy dissipation, $E_{elec}$, increases, the amount of information forwarding at each UN decreases in the same harvest energy case and the average throughput performance of our proposed method will be closer to that of the “without user cooperation” method. In addition, our proposed method is superior to the two traditional methods.

**Figure 9:**
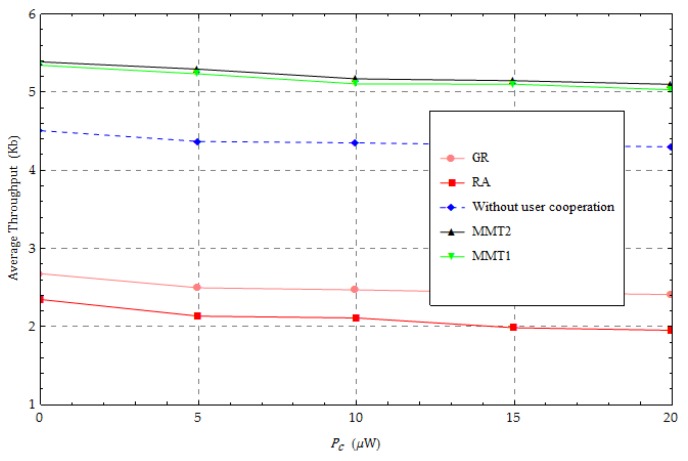
Change in the energy consumption required for transmission.

We further show the effect of the additional circuit loss energy consumption for transmission in Figure 9. As the energy consumption increases, the performances of all methods decrease almost linearly, with very small slopes, because the additional energy consumption has a very low duty cycle in terms of transmission energy consumption. In the transmission process, most of the energy is provided to the UN, i, to transmit signals, that is $P_{c}\ll P_{i},i\in \mathbb{N}$. The average throughput performance when using our proposed method is superior to those of the other methods.

**Figure 10:**
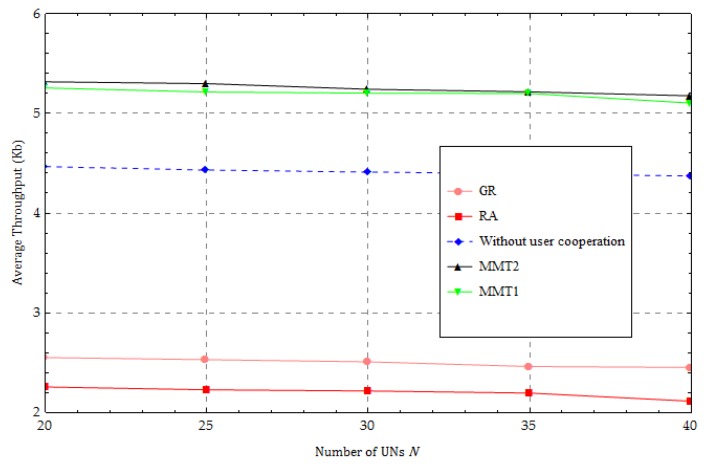
Change in the number of user nodes.

We investigate the average throughput versus the number of users in Figure 10. As the number of UNs increases, the number of forwarding links formed by the UNs increases. More links can help UNs to forward their information and, indirectly, improve the average throughput performance. However, increasing the number of UNs causes less transmission time to be allocated to each UN and leads to a decrease in the average throughput performance. Therefore, the average throughput performances of the all methods remain nearly constant and we find that our method has the best performance.

## 6. Conclusions

In this paper, we proposed a user cooperation transmission method to solve the “near-far” problem in multi-hop WPCNs. Based on the method, we formulated the max-min throughput fairness problem as a mixed non-linear integer programming model. We obtained the upper and lower bounds for the optimal solution by the proposed approximate algorithm based on the piece-wise linear method and performed simulation studies to assess our algorithm. We also showed the impact of system setups to the max-min throughput performance. From simulation results, we showed that the proposed algorithm can obtain the compact upper and lower bounds for the problem in different system setups. Through comparison with three benchmark methods, we also showed the proposed method can effectively enhance the throughput fairness when the transmission power of the HAP is sufficiently large to provide enough energy to user nodes for forwarding information. Furthermore, the proposed method can also effectively improve the max-min throughput performance when the amount of energy consumption of the receiver circuitry or the path-loss exponent is small enough. The proposed method has evident performance gain over the other methods. 

## Appendix A

**Proof** **of** **Lemma** **3.** We use the dual theory to analyze the MMT-UP$\left({\bar{P}}_{i},{\bar{U}}_{ij}\right)$ and MMT-down$\left({\bar{P}}_{i},{\bar{U}}_{ij}\right)$ models. The dual variable, corresponding to the ith constraint in constraint (17), is denoted by $\lambda_{i}$. The dual variable, corresponding to constraint (18), is denoted by μ. Let $\alpha_{i}$ and $\beta_{i}$ denote the dual variables corresponding to the ith constraint in constraints (19) and (21). We denote the dual variable corresponding to the link, $L_{ij}$, in constraints (22) and (23) as $\varepsilon_{ij}$. The dual problems of MMT-UP$\left({\bar{P}}_{i},{\bar{U}}_{ij}\right)$ and MMT-DOWN$\left({\bar{P}}_{i},{\bar{U}}_{ij}\right)$ are denoted by D-MMT-UP$\left({\bar{P}}_{i},{\bar{U}}_{ij}\right)$ and D-MMT-DOWN$\left({\bar{P}}_{i},{\bar{U}}_{ij}\right)$. D-MMT-UP$\left({\bar{P}}_{i},{\bar{U}}_{ij}\right)$ can be expressed as:

$$\min\;\mu +\frac{\gamma W}{ln2}\sum_{L_{ij}\in \bar{L}}\varepsilon_{ij}$$

$$s.t.\;\sum_{i\in V/H}\lambda_{i}\geq 1$$

$$-\lambda_{i}+\beta_{i}\geq 0,\forall i\in \mathbb{N}$$

$$\mu -\sum_{i\in V/H}\alpha_{i}\zeta P_{0}h_{Hi}\geq 0$$

$$\mu +\alpha_{i}(P_{c}+{\bar{P}}_{i})-{\hat{c}}_{ij}\varepsilon_{ij}\geq 0,\forall i\in V/H,\forall L_{ij}\in \bar{L}$$

$$\alpha_{j}E_{elec}+\varepsilon_{ij}+\beta_{j}-\beta_{i}\geq 0,i\in V/H,\forall L_{ij}\in \bar{L}\;$$

where all the dual variables are non-negative. We can also write D-MMT-DOWN$\left({\bar{P}}_{i},{\bar{U}}_{ij}\right)$ as:

min μ

$$s.t.\mathrm{Same}\;\mathrm{constraints}\mathrm{in}\;\mathrm{D-MMT-UP}\left({\bar{P}}_{i},{\bar{U}}_{ij}\right)$$

The above two dual models and their corresponding original problem models are all convex optimization models. We use $\left(\mu^{*},\varepsilon_{ij}{}^{*}\right)$ to denote a part of the optimal solution of the model D-MMT-DOWN$\left({\bar{P}}_{i},{\bar{U}}_{ij}\right)$. Since the above two dual models have the same constraints, $\left(\mu^{*},\varepsilon_{ij}{}^{*}\right)$ is a feasible solution of D-MMT-UP$\left({\bar{P}}_{i},{\bar{U}}_{ij}\right)$. According to convex optimization theory, as the original problem is a maximization problem, any feasible solution of the dual problem is not less than the optimal solution of the original problem. Thus, we have:

$$\bar{f}\leq \mu^{*}+\frac{\gamma W}{ln2}\underset{L_{ij}\in \bar{L}}{\sum }\varepsilon_{ij}{}^{*}$$

The object value of D-MMT-DOWN$\left({\bar{P}}_{i},{\bar{U}}_{ij}\right)$ is same as that of MMT-DOWN$\left({\bar{P}}_{i},{\bar{U}}_{ij}\right)$, that is $\underline{f}=\mu^{*}$. Thus, the following formula is established:

$$\Delta f=\bar{f}-\underline{f}\leq \mu^{*}+\frac{\gamma W}{ln2}\underset{L_{ij}\in \bar{L}}{\sum }\varepsilon_{ij}{}^{*}-\mu^{*}=\frac{\gamma W}{ln2}\underset{L_{ij}\in \bar{L}}{\sum }\varepsilon_{ij}{}^{*}.$$

The dual variable, $\varepsilon_{ij}{}^{*}$, can also be regarded as the corresponding increment in the optimal object value as the constant term of constraint (23) increases by one unit. According to the concept of shadow price, the dual variable, $\varepsilon_{ij}$, is taken as the shadow price of the resource capacity of the link, $L_{ij}$. When the link capacity of $L_{ij}$ is increased by 1, the amount of information the UN, i, can transmit increases by no more than one through link, $L_{ij}$. Thus, $\varepsilon_{ij}{}^{*}\leq 1$. Therefore, the following formula holds:

$$\underset{L_{ij}\in \bar{L}}{\sum }\varepsilon_{ij}{}^{*}\leq \left|\bar{L}\right|$$

From the above inequalities, we obtain:

$$\Delta f\leq \frac{\gamma W}{ln2}\left|\bar{L}\right|.$$

□
